# Effect of Anxiety and Calling on Professional Quality of Life in COVID-19 Dedicated Nurses in Korea

**DOI:** 10.3390/healthcare10091797

**Published:** 2022-09-18

**Authors:** Minjung Moon, Kyoungsan Seo

**Affiliations:** 1 Chungnam National University Hospital & Graduate School in Chungnam National University, Daejeon 35015, Korea; 2 College of Nursing, Chungnam National University, Daejeon 35015, Korea

**Keywords:** anxiety, burnout, COVID-19, nurses, professional quality of life

## Abstract

This study was conducted to investigate the anxiety, calling, and professional quality of life (ProQOL) of coronavirus disease 2019 (COVID-19)-dedicated nurses at COVID-19 hospitals and to identify the factors influencing the ProQOL in COVID-19-dedicated nurses. For this descriptive correlational study, data were collected from June to September, 2021, using structural questionnaires completed by 149 nurses working at four general hospitals with inpatient treatment facilities for patients with COVID-19 in Korea. The State–Trait Anxiety Inventory, Multidimensional Calling Measure, and ProQOL 5 were employed for the survey. The data were examined using descriptive analysis, independent *t*-tests, one-way analysis of variance, Pearson’s correlation coefficients, and multiple regression analysis. The factors influencing compassion satisfaction were state anxiety, trait anxiety, and calling, with an explanatory power of 64%. The factors influencing burnout were trait anxiety and calling, and the explanatory power was 52%. The factors influencing secondary traumatic stress were state anxiety and trait anxiety, and the explanatory power was 23%. Based on the results, lower anxiety and calling influence the ProQOL of COVID-19-dedicated nurses. We propose that programs to raise and maintain ProQOL should be developed and applied.

## 1. Introduction

Coronavirus disease 2019 (COVID-19), which has been a global pandemic since 2020 [1], is a viral respiratory disease caused by severe acute respiratory syndrome coronavirus 2 (SARS-CoV-2). Because of the rapid spread of the disease [2], over 500 million confirmed patients and 6.21 million deaths worldwide have been reported as of 13 April 2022 [3]. Healthcare professionals, including nurses, play critical roles across all fields of screening, treatment, and disinfection to control the transmission of the infectious disease under the prolonged COVID-19 pandemic conditions [4]. Notably, nurses working at hospitals that the government has assigned to treat patients with COVID-19 have taken up diverse roles, including direct care of patients as well as management of the ward environment and patient nutrition, as they serve as the core staff in the frontline against COVID-19 in Korea [5,6,7].

Nurses constantly stay close to patients to provide contact care, which places them at a high risk of COVID-19 owing to the long-term exposure to the risk of infection via droplet or contact transmission [8]. COVID-19-dedicated nurses face heavy workloads under poor working conditions while bearing much responsibility, and consequently, they experience various psychological and physical difficulties related to anxiety, depression, burnout, stress, and insomnia [9,10,11]. During the previous SARS pandemic, nurses reported feeling anxiety, stress, and excessive tension, leading them to refuse to care for patients with new infectious diseases and causing turnover intention [12]. It is thus necessary to gain a better understanding of the hardships faced by frontline nurses during the COVID-19 pandemic and to develop strategies to resolve the difficulties to retain nurses in the field of patient care.

Professional quality of life (ProQOL) refers to the feelings experienced by individuals in a specialized field of providing care to others, which is subcategorized into compassion satisfaction (CS) as a positive factor and burnout (BO) and secondary traumatic stress (STS) as negative factors [13]. CS may curb high CF, thereby contributing to maintaining ProQOL [13]. CS is a positive experience, beyond job satisfaction, which provides the driving force for work continuation [14]. On the other hand, BO is a state of gradual depletion of emotional, psychological, and physical energy due to an excessive level of work, which could reduce work performance, destroy relationships with colleagues, and threaten safety, ultimately reducing the quality of patient care [15]. STS occurs when an individual finds it difficult to separate work and life, as he or she empathizes with the pain of the patients. In healthcare professionals, including nurses, the level of STS increases as they deeply empathize with the painful emotions of the patients [16]. It is necessary to maintain a high level of ProQOL in nurses to ensure job satisfaction and enhance the quality of nursing care [17]. To this end, the positive factors that lead to an increase in the ProQOL of COVID-19-dedicated nurses should be identified and enhanced.

Previous studies on the ProQOL of nurses have mainly focused on oncology-ward nurses [18] and intensive care unit (ICU) nurses [19,20]. With the recent COVID-19 pandemic, studies have investigated the correlation between fatigue and ProQOL in general-ward nurses [21] and reported high levels of BO and CF among COVID-19-ward nurses and emergency-department nurses [22]. Nevertheless, only a few studies [21,22] have focused on the ProQOL of nurses, and there was no study focusing on COVID-19-dedicated nurses only to investigate the level of ProQOL and determine the factors which simultaneously influence their personal and work environment in Korea.

As individuals with a high level of calling tend to feel more rewarded by the work that they do and less frequently experience negative emotions, such as anger, fear, and discomfort, while maintaining a relatively stable mental state [23], a sense of calling may be a motivating factor in the challenging work environment of nursing to reduce the level of burnout and enhance the retention of nurses [24,25]. A sense of calling and positive professionalism have been reported to be motivating factors for COVID-19-dedicated nurses to continue to participate in the nursing of patients with COVID-19 [26,27]. It is therefore necessary to investigate whether a sense of calling affects the ProQOL of COVID-19-dedicated nurses in a difficult work environment. However, studies are lacking on the levels of calling and ProQOL in COVID-19-dedicated nurses, with no such studies having explored the factors that influence the ProQOL of COVID-19-dedicated nurses in South Korea. This study was conducted to fill this gap in the literature.

Thus, this study aimed to determine the level and the relationship between anxiety, calling, and ProQOL and the effects of anxiety, the level of calling, nurses’ characteristics, and the work environment on the level of ProQOL in COVID-19-dedicated nurses. The results will help develop strategies to enhance the ProQOL of COVID-19-dedicated nurses.

## 2. Materials and Methods

### 2.1. Study Design

This study followed a descriptive correlational design to investigate the influence of anxiety and the level of calling on the ProQOL of COVID-19-dedicated nurses.

### 2.2. Participants

The participants of this study comprised COVID-19-dedicated nurses working at government-designated general hospitals for COVID-19 in South Korea. Nurses were provided an explanation of the purpose of the study and signed an informed consent form prior to participation. COVID-19 wards were broadly divided into COVID-19 negative-pressure wards and the COVID-19 ICU for patients requiring treatment such as mechanical ventilation. Depending on specific conditions, certain hospitals integrated these functions into a given ward. In this study, nurses working at screening facilities and emergency departments for COVID-19-suspected patients, nursing managers who did not provide direct patient care in COVID-19 wards, and new nurses with a career length of less than 6 months [28] were excluded.

The sample size was estimated using the G*power program 3.1.9.4 (https://www.psychologie.hhu.de/arbeitsgruppen/allgemeine-psychologie-und-arbeitspsychologie/gpower, accessed on 1 April 2021). The effect size was determined based on the explanatory power reported by a previous study [19]. Eleven potential predictors were used for regression analysis. These included four characteristics previously identified as influencing factors: age, career length, position, and sex; four variables related to the characteristics of the work environment: working department, dispatch status, past experience in nursing patients with emerging infectious diseases, and subjective perception regarding appropriate rewards; and the levels of state anxiety, trait anxiety, and calling. The minimum sample size for the multiple regression analysis, as estimated for conditions of effect size = 0.20, significance level = 0.05, power = 0.95, and 11 predictors, was *n* = 136, which was satisfied in this study.

### 2.3. Instruments

A structured questionnaire was used to investigate the participants’ general characteristics, working conditions, and main variables. The questions included age, sex, marital status, religion, education level, clinical experience, working department, dispatch status, charge position, past experience of nursing patients with emerging infectious diseases, and subjective perception regarding rewards received.

#### 2.3.1. Anxiety

Spielberger et al. [29] developed the State–Trait Anxiety Inventory (STAI); anxiety was measured using the Korean version [30] of the STAI. The tool has two properties: state anxiety (SAI) and trait anxiety (TAI). The STAI consists of 20 questions of the SAI and 20 questions of the TAI. State anxiety indicates the feeling of anxiety in the present situation. Trait anxiety indicates the anxiety experienced in daily life due to unique personal traits. All questions are answered using a 4-point Likert scale, ranging from a score of 1, “Never” or “Rarely”, to a score of 4, “Frequently” or “Always”. For both types of anxiety, the minimum and maximum scores were 20 and 80, respectively, where higher scores indicate higher levels of anxiety. Ten questions on state anxiety and seven questions on trait anxiety required reverse coding. The reliability of the STAI at the time of development was reported as Cronbach’s α = 0.84 [29], and for the Korean version, the reliability for state anxiety was Cronbach’s α = 0.93, and that for trait anxiety was Cronbach’s α = 0.89 [30]. In this study, the reliability for state anxiety was Cronbach’s α = 0.65, and that for trait anxiety was Cronbach’s α = 0.88. In general, more than 40 scores indicated clinically having anxiety, and a score above 44 indicated major anxiety [31].

#### 2.3.2. Calling 

The Multidimensional Calling Measure (MCM-K) was developed by Hagmaier and Abele [32]. Calling was assessed using the Korean version [33] of it. The tool consists of nine questions, each scored on a 6-point Likert scale, from a score of 1, “Not at all”, to a score of 6, “Extremely”, with a minimum score of 9 and a maximum score of 54. Higher scores indicate higher levels of calling. The tool reliability was reported as Cronbach’s α = 0.85 by Hagmaier and Abele [32] while Cronbach’s α = 0.85 for the Korean version and Cronbach’s α = 0.86 in this study.

#### 2.3.3. ProQOL

ProQOL was measured using the Korean ProQOL, freely open version 5.0 (St. Paul, MN, USA), developed by Stamm [13]. The tool consists of two properties: compassion satisfaction (CS) and compassion fatigue (CF), where CF is divided into burnout (BO) and secondary traumatic stress (STS). Among the questions, 10 are related to overall CS, 10 are related to BO, and 10 are related to STS. Each question is answered using a 5-point Likert scale, from a score of 1, “Not at all”, to a score of 5, “Extremely”, with higher scores indicating higher levels of measured CS, BO, and STS. The ProQOL-5 showed high construct validity at the time of tool development, with Cronbach’s α = 0.88 for CS, Cronbach’s α = 0.75 for BO, and Cronbach’s α = 0.81 for STS [13]. The Korean version of ProQOL-5 has also been validated, with Cronbach’s α being 0.93 for CS, 0.75 for BO, and 0.81 for STS [34]. In this study, Cronbach’s α was 0.90 for CS, 0.70 for BO, and 0.75 for STS.

### 2.4. Data Collection and Ethical Considerations

Following the approval of the institutional review board of a single university hospital (IRB File No.: CNUH 2021-05-055), the data for this study were collected from four COVID-19-designated hospitals in two cities in South Korea between 13 June and 10 September 2021. For this cross-sectional survey study, two cities within the same period of time that could be surveyed were conveniently selected, and all government-designated quarantine hospitals in the cities were contacted. With permission from four of the five hospitals in contact, a questionnaire was distributed to all nurses dedicated to patients with COVID-19. The study protocol was approved by the Department of Nursing at each hospital, through which the questionnaire was distributed among COVID-19-dedicated nurses. Taking into account an approximately 10% missing rate, 160 copies of the questionnaire were distributed, and 157 were collected. With the exclusion of eight copies containing incomplete responses, the data from 149 nurses were used in the final analysis.

Prior to the study, the participants were provided an explanation of the study purpose, contents, and procedures, as well as the predicted risks and advantages, and a gift for participation. The written consent form also indicated that they could drop out without any penalty at any time and explained the policies regarding privacy protection, data anonymity, and the use of data only for research purposes. Only individuals who signed the consent form were provided the questionnaire to complete. For blindness, the completed questionnaire with the signed consent form was placed in individual envelopes, sealed and taped, for storage in a designated place until directly retrieved by the investigator. The collected questionnaire and consent forms were stored in a locked cabinet also to ensure data anonymity. The participants were notified of the fact that all data would be safely discarded at the end of the study. The time required to complete the questionnaire was approximately 15 min, and a small gift of appreciation was distributed with the questionnaire.

### 2.5. Data Analysis

The collected data were analyzed using SPSS Window, version 26.0. IBM Corp. (Armonk, NY, USA). Anxiety and ProQOL were analyzed for each subcategory. For the participants’ general characteristics and work environment characteristics as well as the levels of anxiety, calling, and ProQOL, the frequency, percentage, mean, and standard deviation were obtained through descriptive statistics. Additionally, the differences in ProQOL according to the characteristics were analyzed using the *t*-test and one-way analysis of variance, while the Scheffé post hoc test was performed when necessary. The correlations across anxiety, calling, and ProQOL were analyzed through Pearson’s correlation coefficients. Multiple regression analysis was performed to identify the factors influencing ProQOL. 

## 3. Results

### 3.1. General Characteristics and Work Environment Characteristics

The mean age of the participants in this study was 27.7 ± 4.8 years, with 135 female (90.6%) and 127 unmarried (85.2%) individuals. There were 101 nurses without religion (67.8%) and 127 nurses with a bachelor’s degree (85.2%). The mean career length was 4.0 ± 4.5 years, with five charge nurses responsible for both patient care and duty charges (3.4%). The number of nurses per working department was as follows: 68 (45.6%) in COVID-19-dedicated negative pressure wards, 16 (10.7%) in COVID-19-dedicated ICUs, and 65 (43.6%) in integrated-function wards. The number of dispatch nurses was 86 (57.7%), and 103 nurses (69.1%) did not have past experience in nursing patients with emerging infectious diseases. In terms of subjective perception regarding appropriate rewards, 116 nurses (77.9%) responded that they thought the rewards were inappropriate, while 29 nurses (19.5%) responded that they did not know (Table 1).

### 3.2. Levels of Anxiety and Calling and ProQOL

The mean score for state anxiety was 42.64 ± 5.19, and that for trait anxiety was 44.85 ± 7.95. The mean score for calling was 36.75 ± 6.37. For ProQOL, the mean score for each subcategory was as follows: 31.02 ± 5.56 for CS, 27.68 ± 4.19 for BO, and 25.77 ± 4.75 for STS.

### 3.3. Differences in ProQOL According to General Characteristics and Work-Related Characteristics

The differences in the subcategory scores of ProQOL were examined according to the general and work-related characteristics. For CS, significant differences were found according to the working department (F = 6.78, *p* = 0.002), dispatch status (t = 2.49, *p* = 0.014), and position in charge (t = −2.31, *p* = 0.022) (Table 1). The level of CS was relatively higher in COVID-19-dedicated ICU nurses, dispatch nurses, and charge nurses. For BO, as an aspect of CF, significant differences were found according to sex (t = 3.29, *p* = 0.001), working department (F = 6.29, *p* = 0.002), and dispatch status (t = −2.95, *p* = 0.004) (Table 1). The level of BO was relatively higher in female nurses, nurses working in COVID-19-dedicated negative-pressure wards, and in non-dispatch, COVID-19-dedicated nurses. In terms of STS, significant differences were found according to sex (t = 2.20, *p* = 0.029) and the perception of rewards (F = 3.41, *p* = 0.036) (Table 1). The level of STS was relatively higher in female nurses and in nurses who thought that they were not appropriately rewarded.

### 3.4. Correlations among Anxiety, Calling, and ProQOL

For state anxiety, significant correlations were found with CS (r = 0.29, *p* < 0.001) and STS (r = 0.17, *p* = 0.037). For trait anxiety, a negative correlation with CS was found (r = −0.50, *p* < 0.001), whereas significant positive correlations were found with BO (r = 0.66, *p* < 0.001) and STS (r = 0.44, *p* < 0.001). For calling, a significant positive correlation with CS (r = 0.74, *p* < 0.001) and a significant negative correlation with BO (r = −0.47, *p* < 0.001) were detected (Table 2).

### 3.5. Factors Influencing ProQOL

In this study, the factors influencing ProQOL were identified with regression models for each subcategory. Of the variables among the participants’ general and work-related characteristics, those found in the univariate analysis to be associated with ProQOL were entered into the model as independent variables (working department, position, dispatch status, and sex). For the purpose of regression analysis, sex, working department, and dispatch status were converted to dummy-type variables. The variable of position was excluded because of its high correlation with clinical experience, and subjective perception regarding rewards was excluded because the number of participants in a category was less than 5. Factors correlated with the subcategories were entered as independent variables in the multiple regression models. The model for each ProQOL subcategory is described below.

#### 3.5.1. Factors Affecting Compassion Satisfaction

The autocorrelation coefficient for the errors obtained via the Durbin–Watson test of regression hypotheses was 1.82, which is close to 2, which indicates a lack of autocorrelation and satisfies the assumption of independence of residual errors. In the residual plot to test the homogeneity and normality of the error terms, the assumption of homogeneity was verified, and the probability plot (P-P) showed a normal distribution. In the multicollinearity statistics, the tolerance was 0.24–0.94, exceeding 0.1, and the variance inflation factor (VIF) was 1.07–4.124, which was below 10, indicating the absence of multicollinearity. Multiple regression analysis showed that the factors affecting CS were state anxiety (*β* = 0.11, *p* = 0.048), trait anxiety (*β* = −0.26, *p* < 0.001), and calling (*β* = 0.59, *p* < 0.001). The model fitness was statistically significant (F = 36.32, *p* < 0.001), with 64% explanatory power (63% after adjustment) (Table 3). In other words, a high level of state anxiety, a low level of trait anxiety, and a high level of calling were the factors that increased the level of CS.

#### 3.5.2. Factors Affecting Burnout

The autocorrelation coefficient for the errors obtained via the Durbin–Watson test of regression hypotheses was 1.90, which is close to 2, satisfying the assumption of independence of residual errors. In the residual plot to test the homogeneity and normality of the error terms, the assumption of homogeneity was verified, and the P-P showed a normal distribution. In the multicollinearity statistics, the tolerance was 0.24–0.87, exceeding 0.1, and the VIF was 1.15–4.18, less than 10, which indicates the absence of multicollinearity. Multiple regression analysis showed that factors affecting BO were trait anxiety (*β* = 0.53, *p* < 0.001) and calling (*β* = −0.26, *p* < 0.001), with 52% explanatory power (50% after adjustment). The model fitness was statistically significant (F = 25.22, *p* < 0.001), and the factors related to the increase in the level of BO were a high level of trait anxiety and a low level of calling (Table 4).

#### 3.5.3. Factors Affecting Secondary Traumatic Stress

The autocorrelation coefficient for the errors obtained via the Durbin–Watson test of regression hypotheses was 2.16, which is close to 2, satisfying the assumption of the independence of residual errors. In the residual plot testing of the homogeneity and normality of the error terms, the assumption of homogeneity was verified, and the P-P showed a normal distribution. In the multicollinearity statistics, the tolerance was 0.92–0.99, exceeding 0.1, and the VIF was 1.01–1.09, below 10, which indicated the absence of multicollinearity. Multiple regression analysis showed that the factors affecting STS were state anxiety (*β* = 0.17, *p* = 0.017) and trait anxiety (*β* = 0.42, *p* < 0.001), with 23% explanatory power (22% after adjustment). The model fitness was statistically significant (F = 14.59, *p* < 0.001), and the factors increasing the level of STS were a high level of state anxiety and a high level of trait anxiety (Table 5).

## 4. Discussion

This study investigated the anxiety, calling, and ProQOL of nurses in directive caring for patients with COVID-19 only. In this study, we searched the influence of anxiety and calling on the ProQOL of COVID-19-dedicated nurses in South Korea.

The mean score of state anxiety was 42.64, and that of trait anxiety was 44.85 for COVID-19-dedicated nurses. Because a score above 44 indicates major anxiety, the level of anxiety in COVID-19-dedicated nurses in Korea may be interpreted as high. Given the general lack of studies on the level of anxiety in COVID-19-dedicated nurses in South Korea, our results were compared with those of a study in Turkey [11], which reported scores of 46.5–56.4 for state anxiety in COVID-19-dedicated nurses, which was in agreement with this study. The level of anxiety was similar to or lower than that of ICU nurses in South Korea, 47.17 state anxiety and 48.80 trait anxiety [19], and that of emergency-department nurses, 50.48 state anxiety [35]. As the COVID-19 pandemic continued, the anxiety of COVID-19-dedicated nurses remains notably high, although the mechanism of the new infectious disease was defined and vaccines became available. In an additional analysis, trait anxiety was higher in female nurses (t = 3.35, *p* < 0.001) and in nurses in COVID-19-dedicated negative-pressure wards than in those in COVID-19-dedicated ICUs (F = 3.28, *p* = 0.040). In the case of negative-pressure wards in South Korea, the nurses are required to care for conscious patients and provide holistic care for the patients whose family caregivers cannot stay nearby [5,27]. Moreover, they are required to provide care for patients with conditions of varying severity and extensive professional treatments without considering the task load [5,7,8]. It is suggested to explore how various working environments affect nurses’ anxiety in future studies.

The mean score of the calling level was 36.75, which is similar to the score of 36.04 reported for nurses at regional trauma centers in South Korea, which were measured using the same instrument [36]. A sense of calling was also reported as a factor influencing the post-traumatic growth of nurses caring for patients with COVID-19 [37]. In the additional analysis on calling in this study, a significantly high level of calling was exhibited by male nurses (t = −2.25, *p* = 0.026) and charge nurses (t = −2.71, *p* = 0.007). In this study, a high level of calling in charge nurses was mostly attributed to the relatively older age and longer clinical experience as well as to the sense of responsibility conferred by the position, but given the lack of relevant research, further studies should be conducted.

The mean score of ProQOL in COVID-19-dedicated nurses in this study agreed with the scores reported by a study on ProQOL in general nurses in South Korea [21] but deviated from the scores reported by a study on ProQOL in nurses in Spain (39 for CS and 19 for CF) [22]. In Spain, the ProQOL was higher in nurses than in doctors [22], in contrast to a study conducted in South Korea, which reported higher levels of anxiety and insomnia in nurses than in doctors. This may be interpreted as based on the differences in the mean age of the nurses. In the study of Spanish nurses, the mean age was 46 years [22], while the mean age of Korean nurses in previous studies [21] and in this study ranged between 27 years and the early 30s. In previous studies, a high level of BO in COVID-19-dedicated nurses was reported, and it was suggested that efforts should be taken toward the resolution of BO [27]. In further analysis, the level of CS was higher in charge nurses than in staff nurses in agreement with previous findings [21,22]. In this study, charge nurses had higher levels of calling and CS, but in the regression analysis, the charge nurse position did not show a significant influence on CS or other subcategories of ProQOL.

The regression models in this study showed that the factors affecting CS and BO in COVID-19-dedicated nurses were anxiety and level of calling. Specifically, a high level of state anxiety, a low level of trait anxiety, and a high level of calling increased the level of CS. BO was increased by a high level of trait anxiety and a low level of calling, while STS was increased by high levels of state as well as trait anxiety. Because of the lack of studies on factors influencing ProQOL in COVID-19-dedicated nurses, a comparison was made with studies on ICU nurses [19] and emergency-room nurses [34] in South Korea. The results of these studies are in agreement with our findings. On the other hand, including the working department [17,22] and clinical experience [38], which were noted as factors influencing the ProQOL of nurses in previous studies, could not confirm the effect in the regression analysis of this study. In addition, the results are similar to those of the study suggesting trait anxiety as a risk factor of STS [39].

In consideration of the impact of calling on CS and BO, an intervention to reinforce the sense of calling is likely to help COVID-19-dedicated nurses to maintain their ProQOL. As the sense of calling is not an innate factor and may vary according to the situation [32], intervention strategies based on the understanding of factors such as calling could be helpful. However, we should be careful not to impose the ideal of the hero and force a sense of calling on nurses [7].

As global pandemics of new infectious diseases are likely to occur in the future, the supporting system and intervention strategies should be sustainable and updated on the current situation. In a report by Bryant-Genevier et al. [40], 20% of the healthcare professionals providing care for patients with COVID-19 were shown to be unable to take adequate holidays while experiencing mental health problems, such as anxiety, depression, and post-traumatic stress disorder, for which the study suggested the extended scope of personnel and increased flexibility in work hours. In an additional analysis of this study, the level of BO was higher in non-dispatch than in dispatch nurses. This is a significant finding, based on which the current working environment should be amended. Given that South Korea has only 1/3 of the nursing manpower of other Organization for Economic Cooperation and Development countries [5], the ongoing COVID-19 pandemic is presumed to have caused a further increase in BO in COVID-19-dedicated nurses, without adequate shifts of nursing personnel. The results of this study also indicate that 77.9% of the nurses viewed the current level of rewards as inappropriate in relation to the level of their tasks. Likewise, in a study by Zhang et al. [41], financial rewards were suggested and helped reduce the stress and BO in COVID-19-dedicated healthcare professionals. In addition, emotional support and field training should be systematically provided to the dedicated nurses as noted by previous studies [10,41]. Moreover, compared to BO, which arises from cumulative work stress over a long and continuous time, a single exposure could induce traumatic stress [42], which implies the need for regular efforts to analyze and resolve the causes. In a systematic review of studies on STS in mental healthcare professionals [16], the following was suggested to prevent the incidence of secondary traumatic stress: periodic monitoring within mutually supporting relationships, balanced allocation, in-advance training, and an organizational culture of monitoring for and detecting trauma, providing a safe and supportive environment. Considering that a poor work environment has been indicated as a major cause of STS [43] and increased CF [17] in nurses, the work environment for those who care for patients with COVID-19 should be improved and relevant efforts should be taken at the institutional level. In order for those suggestions to be implemented, legal or constitutional support for implementation must be accompanied including the criteria for the allocation of personnel, the maximum work hours and rotation periods at wards dedicated to infectious diseases, and advanced education and training [5].

## 5. Limitations

This study had some limitations. Generalizing the results of this study to all COVID-19-dedicated nurses should be done carefully, as the study involved convenient sampling of a single region in South Korea and collected data via a cross-sectional survey. In the future, a study is needed to compare the results of this study through repeated studies with a larger sample and to understand the long-term effect on the ProQOL of COVID-19-dedicated nurses. Despite these limitations, the significance of this study lies in having first identified the influence of anxiety and level of calling on the ProQOL in Korean nurses.

## 6. Conclusions

Anxiety and calling are the factors influencing ProQOL in COVID-19-dedicated nurses, with 64% and 52% explanatory power for CS and BO, respectively. Additionally, anxiety also influences STS, with 23% explanatory power. Further studies and interventions are suggested to relieve the anxiety and enhance the sense of calling of COVID-19-dedicated nurses in the face of uncertainty of the progression of this new infectious disease. Furthermore, policies should also be developed to ensure appropriate allocation of personnel and enhance the work environment to maintain the nurses’ ProQOL and retention. Further studies should also monitor changes in anxiety, calling, and ProQOL in COVID-19-dedicated nurses at each step of the progression of the new infectious disease, and its long-term impact should be determined.

## Figures and Tables

**Table 1:** Characteristics of Participants and Differences in Professional Quality of Life According to These Characteristics (*N* = 149).

Characteristic	Categories	*n* (%) or M ± SD (Range)	Professional Quality of Life					
			Compassion Satisfaction		Burnout		Secondary Traumatic Stress	
			M ± SD	F/t *(p)* or r *(p)*	M ± SD	F/t *(p)* or r *(p)*	M ± SD	F/t *(p)* or r *(p)*
Age (years)		27.7 ± 4.8 (22–47)	31.02 ± 5.56	0.09 (0.299)	27.68 ± 4.19	0.01 (0.869)	25.77 ± 4.75	−0.01 (0.862)
Clinical experience (years)		3.99 ± 4.51 (1–23)	31.02 ± 5.56	0.09 (0.303)	27.68 ± 4.19	0.05 (0.532)	25.77 ± 4.75	−0.01 (0.878)
Sex	Female	135 (90.6)	30.78 ± 5.49	−1.66 (0.099)	28.04 ± 4.05	3.29 (0.001)	26.04 ± 4.81	2.20 (0.029)
	Male	14 (9.4)	33.36 ± 5.87		24.29 ± 4.21		23.14 ± 3.30	
Marital status	Married	22 (14.8)	32.18 ± 5.26	1.06 (0.291)	27.32 ± 3.52	−0.44 (0.659)	25.50 ± 4.68	−0.29 (0.773)
	Unmarried	127 (85.2)	30.82 ± 5.61		27.75 ± 4.31		25.82 ± 4.78	
Religion	Yes	48 (32.2)	30.94 ± 6.17	−0.13 (0.901)	27.79 ± 4.48	0.21 (0.831)	26.21 ± 5.23	0.77 (0.442)
	No	101 (67.8)	31.06 ± 5.29		27.63 ± 4.08		25.56 ± 4.53	
Education	Associate	11 (7.4)	29.73 ± 7.01	0.94 (0.395)	26.73 ± 3.13	0.91 (0.406)	24.45 ± 5.29	0.49 (0.612)
	Bachelor	127 (85.2)	30.97 ± 5.40		27.65 ± 4.27		25.84 ± 4.59	
	≥Master	11 (7.4)	32.91 ± 5.97		29.09 ± 4.13		26.27 ± 6.24	
Working department (quarantine only)	COVID-19 isolation ward	68 (45.6)	29.42 ± 5.24	6.78 (0.002) a < b ^†^	28.91 ± 3.90	6.29 (0.002) a > b ^†^	26.35 ± 5.08	1.54 (0.219)
	COVID-19 ICU	16 (10.7)	34.19 ± 5.69		25.69 ± 3.36		24.13 ± 4.01	
	Integrated ward	65 (43.6)	31.92 ± 5.40		26.89 ± 4.34		25.57 ± 4.52	
Dispatch within facility	Yes	86 (57.7)	31.98 ± 5.25	2.49 (0.014)	26.84 ± 3.75	−2.95 (0.004)	25.21 ± 4.28	−1.70 (0.092)
	No	63 (42.3)	29.71 ± 5.75		28.84 ± 4.51		26.54 ± 5.27	
Position in charge	Staff nurse	144 (96.6)	30.83 ± 5.50	−2.31 (0.022)	27.71 ± 4.25	0.37 (0.712)	25.83 ± 4.75	0.85 (0.399)
	Charge nurse	5 (3.4)	36.60 ± 4.61		27.00 ± 1.87		24.00 ± 5.00	
Past experience in nursing patients with EID	Yes	46 (30.9)	31.80 ± 5.96	1.15 (0.252)	26.85 ± 4.19	−1.64 (0.104)	25.04 ± 4.49	−1.25 (0.213)
	No	103 (69.1)	30.67 ± 5.37		28.06 ± 4.16		26.10 ± 4.85	
Subjective perception regarding reward	Appropriate	4 (2.7)	32.00 ± 5.71	0.09 (0.915)	24.50 ± 6.02	2.00 (0.139)	20.50 ± 4.20	3.41 (0.036) a < b ^†^
	Not appropriate	116 (77.9)	30.94 ± 5.69		27.99 ± 4.08		26.16 ± 4.43	
	Do not know	29 (19.5)	31.21 ± 5.18		26.90 ± 4.28		24.93 ± 5.64	

^†^ Scheffé test was performed for post hoc comparisons. EID = emerging infectious diseases; ICU = intensive care unit; M = mean; SD = standard deviation.

**Table 2:** Correlation Among Anxiety, Anxiety, Calling, and ProQOL.

Variables	State Anxiety	Trait Anxiety	Calling	Compassion Satisfaction	Burnout
	r (*p* Value)				
**Trait Anxiety**	0.01 (0.953)	1			
**Calling**	0.28 (0.001)	−0.36 (<0.001)	1		
**Compassion Satisfaction**	0.29 (<0.001)	−0.50 (<0.001)	0.74 (<0.001)	1	
**Burnout**	−0.06 (0.479)	0.66 (<0.001)	−0.47 (<0.001)	−0.64 (<0.001)	1
**Secondary Traumatic Stress**	0.17 (0.037)	0.44 (<0.001)	−0.07 (0.421)	−0.11 (0.170)	0.54 (<0.001)

**Table 3:** Regression Analysis of Factors Affecting Compassion Satisfaction.

	B	Standard Error	*β*	t	*p* Value	*Tolerance*	Variation Inflation Factor
(constant)	18.01	3.89		4.63	<0.001		
State anxiety	0.11	0.06	0.11	1.99	0.048	0.89	1.13
Trait anxiety	−0.18	0.04	−0.26	−4.71	<0.001	0.83	1.21
Calling	0.51	0.05	0.59	10.14	<0.001	0.76	1.33
Working department_ isolation ward *	−2.03	1.14	−0.18	−1.78	0.077	0.24	4.12
Working department_ integrated ward *	−0.75	0.97	−0.07	−0.77	0.440	0.34	2.97
Dispatch status_No	−0.07	0.76	−0.01	−0.09	0.931	0.55	1.81
Position_charge nurse	−1.28	1.60	−0.04	−0.80	0.426	0.94	1.07

*R*^2^ = 0.64, adjusted *R*^2^ = 0.63, F = 36.32, *p* < 0.001. Reference group: Working department * Intensive Care Unit; Dispatch status * Yes; Position * Staff nurse.

**Table 4:** Regression Analysis of Factors Affecting Burnout.

	B	Standard Error	*β*	t	*p* Value	*Tolerance*	Variation Inflation Factor
(constant)	20.23	2.64		7.68	<0.001		
Trait anxiety	0.28	0.03	0.53	8.12	<0.001	0.82	1.23
Calling	−0.17	0.04	−0.26	−4.09	<0.001	0.86	1.17
Working department_ isolation ward *	0.80	1.00	0.10	0.80	0.425	0.24	4.18
Working department_ integrated ward *	0.23	0.85	0.03	0.27	0.790	0.34	2.95
Dispatch status_No	0.64	0.66	0.08	0.96	0.340	0.55	1.81
Sex_Female *	0.58	0.90	0.04	0.65	0.516	0.87	1.15

*R*^2^ = 0.52, adjusted *R*^2^ = 0.50, F = 25.22, *p* < 0.001. Reference group: Working department * Intensive Care Unit; Dispatch status * Yes; Sex * male.

**Table 5:** Regression Analysis of Factors Affecting Secondary Traumatic Stress.

	B	Standard Error	*β*	t	*p* Value	*Tolerance*	Variation Inflation Factor
(constant)	6.39	3.56		1.80	0.075		
State anxiety	0.16	0.07	0.17	2.40	0.017	0.99	1.01
Trait anxiety	0.25	0.05	0.42	5.58	<0.001	0.93	1.08
Sex_Female *	1.33	1.23	0.08	1.08	0.283	0.92	1.09

*R*^2^ = 0.23, adjusted *R*^2^ = 0.22, F = 14.59, *p* < 0.001. Reference group: Sex * male.

## Data Availability

The data are not publicly available because of privacy or ethics.
